# The Role of Vitamin D Metabolism Genes and Their Genomic Background in Shaping Cyclosporine A Dosage Parameters after Kidney Transplantation

**DOI:** 10.3390/jcm13164966

**Published:** 2024-08-22

**Authors:** Katarzyna Kotowska, Bartosz Wojciuk, Jerzy Sieńko, Anna Bogacz, Iga Stukan, Sylwester Drożdżal, Bogusław Czerny, Karol Tejchman, Grzegorz Trybek, Bogusław Machaliński, Maciej Kotowski

**Affiliations:** 1 Clinic of Maxillofacial Surgery, Pomeranian Medical University in Szczecin, 70-111 Szczecin, Poland; 2 Department of Immunological Diagnostics, Pomeranian Medical University in Szczecin, 70-111 Szczecin, Poland; 3 Institute of Physical Culture Sciences, University of Szczecin, 70-453 Szczecin, Poland; 4 Department of Personalized Medicine and Cell Therapy, Regional Blood Center, 60-354 Poznan, Poland; 5 Department of General Pathology, Pomeranian Medical University in Szczecin, 70-111 Szczecin, Poland; 6 Department of Nephrology, Transplantology and Internal Medicine, Pomeranian Medical University in Szczecin, 70-111 Szczecin, Poland; 7 Department of General Pharmacology and Pharmacoeconomics, Pomeranian Medical University in Szczecin, 71-210 Szczecin, Poland; 8 Department of General Surgery and Transplantation, Pomeranian Medical University in Szczecin, 70-111 Szczecin, Poland; 9 Department of Interdisciplinary Dentistry, Pomeranian Medical University, 70-204 Szczecin, Poland

**Keywords:** chronic kidney disease, vitamin D, calcineurin inhibitor, cyclosporin A, classification and regression trees, data mining

## Abstract

**Background:** Kidney transplantation is followed by immunosuppressive therapy involving calcineurin inhibitors (CNIs) such as cyclosporin A. However, long-term high CNIs doses can lead to vitamin D deficiency, and genetic variations influencing vitamin D levels can indirectly impact the necessary CNIs dosage. This study investigates the impact of genetic variations of vitamin D binding protein (*DBP*) rs2282679 and *CYP2R1* hydroxylase rs10741657 polymorphisms on the cyclosporin A dosage in kidney transplant recipients. Additional polymorphisims of genes that are predicted to influence the pharmacogenetic profile were included. **Methods:** Gene polymorphisms in 177 kidney transplant recipients were analyzed using data mining techniques, including the Random Forest algorithm and Classification and Regression Trees (C&RT). The relationship between the concentration/dose (C/D) ratio of cyclosporin A and genetic profiles was assessed to determine the predictive value of *DBP* rs2282679 and *CYP2R1* rs10741657 polymorphisms. **Results:** Polymorphic variants of the *DBP* (rs2282679) demonstrated a strong predictive value for the cyclosporin A C/D ratio in post-kidney transplantation patients. By contrast, the *CYP2R1* polymorphism (rs10741657) did not show predictive significance. Additionally, the immune response genes rs231775 *CTLA4* and rs1800896 *IL10* were identified as predictors of cyclosporin A response, though these did not result in statistically significant differences. **Conclusions:** *DBP* rs2282679 polymorphisms can significantly predict the cyclosporin A C/D ratio, potentially enhancing the accuracy of CNI dosing. This can help identify patient groups at risk of vitamin D deficiency, ultimately improving the management of kidney transplant recipients. Understanding these genetic influences allows for more personalized and effective treatment strategies, contributing to better long-term outcomes for patients.

## 1. Introduction

Chronic Kidney Disease (CKD) affects 100 million people in Europe and is projected to become the fifth leading cause of death worldwide by 2040. The actual number of patients may be even higher, as many remain unaware of the disease due to the lack of early diagnosis. Furthermore, CKD is one of the most costly diseases for healthcare systems, with an estimated annual expenditure of EUR 140 billion in Europe.

As the disease progresses, renal replacement therapy is frequently required, with kidney transplantation representing the optimal treatment, accompanied by the essential inclusion of immunosuppressive therapy. Calcineurin inhibitors (CNIs) were introduced into clinical practice in the early 1980s and quickly became the cornerstone of immunosuppressive therapy following organ transplantation. The first drug of this class to be introduced was cyclosporin A (CsA). At present, the first-line immunosuppressive drug is tacrolimus, which forms the basis of immunosuppressive regimens. However, tacrolimus and other immunosuppressants may induce vitamin D deficiency [1,2].

The latter presents a serious problem in transplant recipients [3,4]. The main causes of vitamin D deficiency in this group include the impaired uptake of calcidiol by the transplanted kidney, the indirect effects of immunosuppressive drugs, hyperparathyroidism, and disruptions to the calcium and phosphate balance [5]. This issue arises because vitamin D has a pleiotropic effect, ranging from the skeletal system to the regulation of over 900 genes involved in innate and adaptive immunity [6,7,8,9,10,11,12]. In kidney transplant recipients, the complications stemming from vitamin D deficiency pose a significant concern regarding alterations in the skeletal system [4]. At the same time, vitamin D deficiency is associated with dysregulated immune responses, the development of insulin resistance, vascular dysfunction and damage, and cardiomyopathy. Hesketh et al. demonstrated that vitamin D helps to modulate immunosuppression [13]. Other studies have linked vitamin D deficiency to an increased risk of kidney transplant failure [14,15].

In humans, the major source of vitamin D is the cutaneous synthesis of cholecalciferol (vitamin D) following exposure to ultraviolet B (UVB) radiation during sun exposure, which is influenced by body temperature. The next step is 25-hydroxylation, which occurs in the liver primarily by P450 2R1 25-hydroxylase (CYP2R1), resulting in the synthesis of calcidiol. Polymorphisms in the *CYP2R1* gene are associated with variable 25-hydroxylase activity, which affects serum 25 (OH)D levels [16]. The serum concentration of calcidiol strongly correlates with the single nucleotide polymorphism rs2282679 A>C in the *GC* gene. The *GC* gene encodes for vitamin D binding protein (DBP)—a protein crucial for calcidiol and calcitriol transportation. In the renal tubules, CYP27B1 1α-hydroxylase mediates a secondary hydroxylation that produces the active form of vitamin D—calcitriol. Calcitriol can bind intracellularly to the nuclear vitamin D receptor (VDR) and thus exert its target effect [17]. CYP3A4 polymorphisms influence the pharmacokinetic mechanisms involved in the metabolism of both tacrolimus and cyclosporine A [18,19,20].

Genetic variability due to multiple polymorphisms may alter the bioavailability and modulate the effects of vitamin D metabolites. For instance, vitamin D supplementation can affect serum levels differently depending on the genetic variant of the *CYP2R1* gene in a healthy individual [16,21]. Cyclosporin A (CsA) and tacrolimus inhibit hepatic CYP3A4 (25-hydroxylase activity), resulting in reduced vitamin D levels [2].

In exploring the interplay between CNIs and vitamin D activity at different stages, we aimed to investigate the association of two major vitamin D polymorphisms with parameters of cyclosporine A dosage. The main objective of our study was to examine the influence of the polymorphisms rs10741657 (CYP2R1) and rs2282679 (GC) on the cyclosporine A C/D ratio, while considering other selected genomic factors potentially associated with calcineurin inhibitor pharmacodynamics. The ontology of other genes involved in our analysis pertained to the immune response and hepatic drug metabolism. Additionally, we sought to develop a predictive algorithm to optimize cyclosporine A dosing. Previous studies have demonstrated success with similar approaches in various post-transplantation patient populations [22,23,24,25,26].

Given our genetic database comprising a large, homogeneous patient cohort and considering the growing, yet unclear, body of information on the influence of vitamin D on CNI therapy, we found it valuable to employ data mining techniques to explore this field.

## 2. Materials and Methods

The patients were recruited at the Department of Nephrology and Kidney Transplantation of the Independent Public Provincial Hospital in Szczecin and the Department of General and Transplant Surgery of the Pomeranian Medical University in Szczecin. The research was approved by the Bioethics Committee of the Medical University of Karol Marcinkowski in Poznań—resolution No. 560/17. All patients signed an informed consent form.

Only patients with stable graft function who were undergoing routine follow-up at the outpatient clinic were included in the study. Patients with unstable graft function who required hospitalization were excluded from the study group.

### 2.1. Selection and Characteristics of the Study Group

The study cohort consisted of 177 patients of both genders, ranging in age from 18 to 80, who had undergone kidney transplantation and were receiving cyclosporine (CsA), a calcineurin inhibitor, as part of their immunosuppressive therapy. To assess the immunosuppressant concentration, blood samples were collected from the bend of the elbow vein before the morning drug dose administration for each patient in a fasting state. A brief overview of the characteristics of the study group is presented in Table 1 and Table 2.

The genetic study of the polymorphisms was carried out on the abovementioned biological material, which was stored at a temperature of −20 °C.

### 2.2. Isolation of Samples

Genomic DNA isolation from blood samples was performed using the QIAamp Mini Blood kit (Qiagen, Hilden, Germany) according to the manufacturer’s protocol. The purity and concentration of each DNA sample were assessed using spectrophotometry (EPOCH Spectrophotometer, Agilent Technologies, Santa Clara, CA, USA). Targeted polymorphisms were analyzed by real-time PCR using LightCycler^®^ 480 (Roche Diagnostics, Rotkreuz, Switzerland). For genotyping, hybridization probes were used as fluorescent dyes labeled with SimpleProbe (Roche, Basel, Switzerland). The results were analyzed based on the melting curve using the LightCycler^®^ 480 Basic Software (16 February 2023). The LightSNiP rs2282679 and rs10741657 collection of polymorphisms contains the appropriate concentrations of specific primers and probes for the amplified DNA fragments, prepared according to the manufacturer’s instructions. The concentration of CsA in the whole blood was determined using the microparticle chemiluminescent immunoassay (CMIA) (ARCHITECT i2000SR analyzer, Abbott laboratories, Green Oaks, IL, USA). The C/D ratio (concentration/dose ratio) was calculated for each patient by dividing the values of the detected blood concentration to the applied dose.

The study of polymorphisms was carried out in the Laboratory of Experimental Pharmacogenetics of the Department of Clinical Pharmacy and Biopharmacy and the Department of Stem Cells and Regenerative Medicine of the Institute of Natural Fibers and Medicinal Plants of the Medical University of Poznań. We involved the data on 16 polymorphisms identified in 15 genes. A complete list of the genes and polymorphisms analyzed is presented in Table 3.

The genetic database used for this analysis was developed gradually over several years. Polymorphisms were selected based on their possible pharmacogenetic or pathophysiological impact on the metabolism of CNI drugs or related processes involving the immune response.

### 2.3. Statistical Analysis

The analysis of the relationship between the C/D ratio and the genetic profile was performed using data mining methodology. The Random Forest algorithm was used, followed by Classification and Regression Trees (C&RT) in Statistica software (ver. 13.1, StatSoft Polska, Cracow, Poland). The Random Forest analysis is frequently used in pharmacology research and was successfully employed by others [22,24,27,28,29,30,31]. The analysis was supplemented with ANOVA tests together with Tukey’s post-hoc test for various n, in the case of variables satisfying the condition of normal distribution. The Shapiro–Wilk test was used to assess the normality of the distribution.

## 3. Results

In the first stage, we performed an analysis using the Random Forest algorithm to determine the importance of individual predictors. Random Forest analysis for CsA revealed the 49A>G RS231775 polymorphism in the *CTLA4* gene as the highest rank predictor. The rs2282679GC polymorphisms of the vitamin D binding protein gene and the rs10741657 of CYP2R1 25-hydroxylase gene polymorphisms revealed similar, lower ranks.

**Table 3:** Importance of individual predictors for CsA. Random Forest analysis of DNA samples isolated from the peripheral blood of patients post-kidney transplantation treated with cyclosporin A as an immunosuppressant revealed that the rs2282679GC polymorphism had a high predictive rank, while the rs10741657 polymorphism of the *CYP2R1* 25-hydroxylase gene showed a similar but lower predictor importance.

Polymorphism	Rank ^1^
rs231775 CTLA4	100
rs602662 FUT2	94
rs2282679 GC	90
rs1800896 IL10	89
rs1800795 IL6	88
rs8175347 UGT1A1*28	86
rs4654748 NBPF3	83
rs2032582 MDR	80
rs1045642 MDR	79
rs2069762 IL2	76
rs10741657 CYP2R1	72
rs1800468 TGFB	63
rs1800629 TNF-alfa	53
rs12272004	53
rs33972313 SLC23A1	41
rs2740574 CYP3A4	22

^1^ Predictor importance for CsA with C/D ratio as a dependent variable.

In the next stage, the Classification and Regression Trees (C&RT) algorithm was used to create binary regression models for cyclosporine A C/D ratio values. The hierarchy of individual ranks obtained from the Random Forest algorithm was considered. Predictors with importance values of less than 0.7 for CsA were excluded. A 10-fold cross-validation was applied. The following validation parameters were used: the cost of resubstitution, the cost of cross-check, and the standard error.

**Figure 1:**
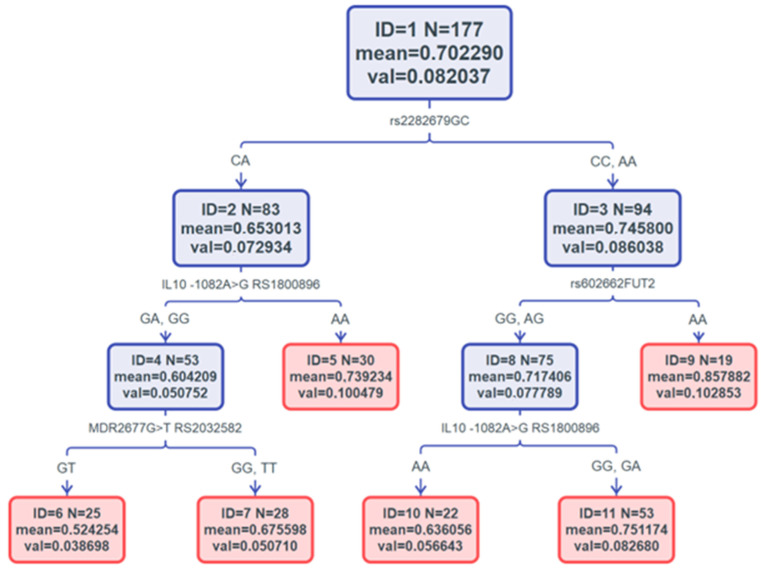
C&RT regression decision tree model for C/D ratio of cyclosporin A and the analyzed SNP. Model parameters: node count: 11; knot counts 15; trim measure: variance; standard error: 0.01; resubstitution cost 0.07; SK cost 0.09. Terminal leaves are marked in red, and four of these are characterized with at least one SNP. Leaves’ IDs, mean and variance values are reported inside the leaves and nodes. The genotypes relevant for particular leaves are described below.

**Figure 2:**
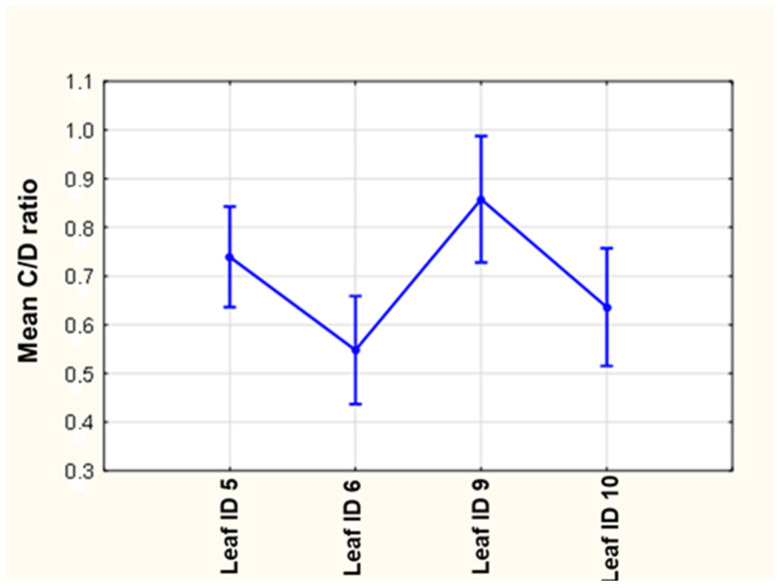
ANOVA results for C/D CsA. ANOVA analysis of the mean value of C/D ratio ±95% confidence interval belonging to the final four leaves of the regression model shown in Figure 1. The results show the statistically significant difference between mean C/D ratio value of recipients with polymorphisms: leaf ID 10: rs2282679 CC or AA, rs602662 FUT2 GG or AG, IL-10-1082A>G RS1800896 AA; and leaf ID 9: rs2282679 CC or CA, rs602662 FUT2 AA, *p* = 0.006. Statistical analysis was performed using Statistica software (ver. 13.1).

Figure 1 shows the regression model for the C/D CsA values. The following attributes were included in the analysis: rs231775 *CTLA4*, rs602662 *FUT2*, rs2282679 *GC*, rs1800896 *IL10*, rs1800795 *IL6*, rs8175347 UGT1A1*28, rs4654748 *NBPF3*, rs2032582 *MDR*, rs1045642 *MDR2*, and rs1045642 *MDR*. Detailed tree characteristics and validation parameters are presented in Figure 2. The model revealed the rs2282679 *GC* polymorphism as an overarching predictor and a series of leaves described by the mean C/D value and coexisting polymorphisms. The rs10741657 *CYP2R1* polymorphism was not identified as a predictor in the model. The mean C/D values of individual leaves and nodes are shown in boxes. Considering the binary nature of the tree, four final leaves were distinguished qualitatively, characterized by at least one specific polymorphism. The genetic characteristics of all four abovementioned leaves is listed below.

1. Leaf ID 6: rs2282679 CA, IL 10-1082A>G RS1800896 GA or GG, MDR2677G>T RS2032582 GT
2. Leaf ID 5: rs2282679 CA, IL10-1082A>G RS1800896 AA
3. Leaf ID 10: rs2282679 CC or AA, rs602662 *FUT2* GG or AG, *IL10*-1082A>G RS1800896 AA
4. Leaf ID 9: rs2282679 CC or CA, rs602662 *FUT2* AA

IL101082A>GRS1800896 AA appeared as a defining single polymorphism for two of the leaves (ID 5 and ID 10). However, MDR2677G>Trs2032582 (leaf ID 6) and rs602662FUT2 (leaf ID 9) were decisive for statistical significance in cyclosporine C/D ratios (*p* = 0.006, Figure 2 and Table 4).

## 4. Discussion

In the presented study, using the Random Forest method, it was shown that the studied polymorphisms, which have a strong influence on vitamin D metabolism, are associated with a high predictive rank for the C/D ratio of cyclosporine. The significance of the rs2282679 GC polymorphism was then confirmed in the C&RT model.

There is currently no reliable data in the literature regarding the variability of effective drug dosage over time, which may be influenced by factors such as epigenetic changes. Considering this, the concentration/dose ratio, which is regarded as the most independent measure of therapy efficacy, was used for the analysis in the presented study.

The GWAS study on a large cohort of subjects confirmed the significance of the polymorphisms rs2282679, rs12785878, and rs10741657 in relation to serum concentrations of 25 (OH) D [32,33,34]. It was suggested that certain variants might serve as serum deficiency predictors [35]. Homozygosity for the C allele in rs2282679 has been associated with lower serum concentrations of vitamin D compared to AC heterozygosity and AA homozygosity [32,35]. Consequently, this has been found to increase the risk of vitamin D deficiency and a suboptimal response to vitamin D supplementation [36], in both adults and children, irrespective of the season [37]. Perna et al. showed that carriers of at least one copy of the less frequent allele exhibit a lower efficiency of skin vitamin D synthesis [38]. Dong et al. reported that rs2282679 alleles negatively affect serum vitamin D levels in pregnant women. Using decision tree methodology, they created a model to assess the risk of deficiency, and based on the results, the clinical procedure was modified [39]. This study effectively integrates the results of genetic determinations with data mining methods, which ultimately become tools of clinical significance.

Similarly to the rs2282679 *GC*, the rs10741657 *CYP2R1* polymorphism is associated with vitamin D serum concentration. It is believed to regulate gene expression and, therefore, may influence the level of 25-hydroxylase activity and expression. Based on genome-wide association studies by Ahn et al. [40], Anderson et al. [41], O’Brien et al. [42], and Jiang et al. [43], more than 20 SNPs of the *CYP2R1* gene were found to affect vitamin D concentration in the European population. Carriers of two G alleles in rs10741657 are characterized by significantly lower vitamin D concentrations compared to AG heterozygotes and AA homozygotes. The GG genotype shows decreased *CYP2R1* enzyme activity, which translates into a marked reduction in 25 (OH) D concentrations compared to the AA genotype. In several populations, carriers of the G allele exhibited the lowest serum concentrations of 25 (OH) D, demonstrating that the tested SNP correlates with 25 (OH) D levels. By contrast, the AA and AG genotypes of *CYP2R1* rs10741657 are associated with a greater increase in 25 (OH) D after UVB exposure compared to GG homozygotes [43]. The relationship between rs10741657 and the increased risk of vitamin D deficiency was significant in the dominant model (GG + AG/AA), but not in the recessive model (GG/AG + AA) [44].

Vitamin D deficiency due to genetic variation can be effectively addressed through supplementation, and genotyping can aid in identifying at-risk groups [45]. Many studies investigating the association between genetic variation and vitamin D concentration focus on polymorphisms that affect the functioning of DBP and vitamin D hydroxylases [46]. Our study also addresses this issue. The analysis using the Random Forest algorithm confirmed that both studied polymorphisms (rs2282679 *GC* and rs10741657 *CYP2R1*) had a high predictive rank with respect to the C/D ratio in patients after kidney transplantation. However, the C&RT algorithm analysis did not confirm the predictive significance of the rs10741657 *CYP2R1* polymorphism in the tested model; therefore, the discussion will focus on the role of the rs2282679 *GC* polymorphism.

Kidney transplantation is the gold standard in the treatment of end-stage renal disease (ESRD), providing patients with statistically longer survival compared to other renal-replacement therapies. Any improvements that enhance and prolong organ function and improve the quality and length of life for recipients are of particular interest. One of the methods proposed in this work is the genotyping of polymorphisms involved in the vitamin D biosynthesis pathway as a significant factor modulating recipients’ homeostasis. Our results, supported by studies conducted in various centers, demonstrate that the *GC* polymorphism (rs2282679) in the gene for vitamin D binding protein impacts therapy with calcineurin inhibitors.

There is a relationship between rs2282679 DBP polymorphism and carcinogenesis. As mentioned, the C allele, in comparison to AA homozygotes, increases the risk of a reduced DBP concentration and vitamin D deficiency. It has also been shown that DBP levels are associated with a shorter interval between relapses in colorectal cancer and an increased risk of several other cancers [47,48,49]. DBP is a precursor molecule for a potent macrophage activating factor, and AA homozygotes exhibit higher serum DBP concentrations, which may exert a more substantial immunomodulatory effect than in the AC/CC genotypes. These data could be relevant for transplantation patients with an increased cancer risk [50].

Moreover, the rs2282679 polymorphism and lower levels of vitamin D are associated with greater disease severity and a higher risk of death in patients with SARS-CoV-2 infection. These results demonstrate that DBP plays an active role in inflammation, including a chemotactic effect on neutrophils and the activation of macrophages. In addition, DBP has a protective effect on thromboembolic complications during COVID-19 [51].

There is also a significant correlation between DBP concentration and bone mineral density (BMD). While low BMD is not a disease marker per se, it reflects overall bone health. A healthy skeletal and muscular system determines the maintenance of mobility—an important factor in preventing chronic diseases. Low serum DBP is associated with disturbed calcium homeostasis, decreased bone mineral density, and an increased incidence of osteoporotic fractures, particularly in postmenopausal women [52]. Postmenopausal recipients require special attention, as reduced concentrations of vitamin D and DBP are often observed in this patient group [53,54]. Genotyping could contribute to the development of individualized and more effective treatment plans, thereby reducing the risk of complications related to calcium and phosphate metabolism disorders [55]. DBP may also influence BMD through another mechanism: by serving as a reservoir for vitamin D metabolites, suggesting that DBP can regulate their bioavailability and the half-life of vitamin D [52]. The association of the two major *GC* variants with serum DBP and BMD has been confirmed [52]. Therefore, identifying a sensitive and predictive biomarker for detecting osteoporosis would benefit patients.

The work of Ponticelli and Passerini described numerous gastrointestinal complications in patients after kidney transplantation, which may lead to the loss of the transplanted organ [56]. In the context of kidney transplantation, immunosuppressive therapy can disrupt the intestinal immune environment, increasing susceptibility to damage. This disruption compromises the epithelial barrier of the intestinal mucosa, allowing prolonged exposure to antigens. Calcitriol enhances the intestinal barrier by stimulating the expression of tight junction proteins between enterocytes [57,58]. The loss of the intestinal barrier function is also thought to contribute to the development of GvHD. The vitamin D-dependent release of cathelicidin and the protection of epithelial barriers (including those in other parts of the digestive and genitourinary tracts) may affect the transplanted organ’s functionality during infection. Furthermore, vitamin D inhibits inflammatory mediators released by neutrophils, such as TNF-α, IL-6, and IL-8 [59].

Vitamin D and VDR may regulate the synthesis of endothelial nitric oxide (NO) [60]. Nitric oxide has a vasodilating effect, which protects blood vessels. Vitamin D supplementation may reduce endothelial dysfunction, as confirmed by long-term studies [61,62,63,64,65]. Adequate levels of vitamin D have a protective effect under oxidative stress conditions, which can occur during transplantation [66]. In addition, cyclosporin A and tacrolimus inhibit NO synthesis, leading to a reduced endothelial response to NO and a secondary lack of renal vasodilation [67,68]. There is also an increased risk of post-transplant diabetes mellitus (PTDM) associated with low vitamin D levels and impaired glucose metabolism [69]. It has been shown that pancreatic β cells express the enzyme 1α-hydroxylase and vitamin D receptors, which may influence insulin secretion [70].

The immunomodulatory properties of vitamin D affect the doses of immunosuppressants used by organ recipients [71]. Following heart transplantation, patients receiving low-dose calcitriol required significantly lower cumulative doses of cyclosporin A without affecting the incidence of rejection episodes, infection, or death [23]. Vitamin D enhances the expression of the *CYP3A4* gene. Lindh et al. showed that this effect varies with the season, as vitamin D concentration is strongly related to insolation. Furthermore, as reported, individual changes in vitamin D levels may influence the metabolism of drugs including cyclosporin A, tacrolimus, and sirolimus. Notably, the authors also used the C/D ratio for their analysis [71]. Vitamin D affects the expression of genes dependent on it through its receptor [72]. The effect of vitamin D on *CYP3A4* transcription is related to its interaction with a specific VDR receptor [71,73]. Therefore, it is possible that the CYP3A4 cytochrome might be the key to understanding the interaction between vitamin D and CNIs [74]. Both vitamin D and calcineurin inhibitors are CYP3A4 substrates. Additionally, vitamin D has a cytochrome-inducing effect [75,76,77]. In our study, however, the polymorphism rs2740574 *CYP3A4* had a very low predictive rank for the cyclosporin A C/D ratio.

Most immunosuppressants have a narrow therapeutic range. The occurrence of most side effects is dose-dependent and correlates with the drug concentration in the blood serum. The main goal in developing immunomodulatory strategies is to establish drug combinations that produce synergistic effects while minimizing toxicity. There is a synergistic relationship between vitamin D function and immunosuppressive therapy [78,79,80,81,82,83]. Notably, Gim et al. analyzed the effect of the variability of rs776746 (*CYP3A5*) and rs1137115 (*CYP2A6*) on tacrolimus dosing using data mining methods. They emphasized that combining decision tree algorithms with Random Forest algorithms is an effective tool for pharmacogenetic analysis [84]. The decision tree algorithm has been shown to outperform other data mining methods [26].

In our study, rs2282679GC demonstrated a superior predictive capability regarding cyclosporine A dosage parameters compared to other polymorphisms. Interestingly, immune response-related genes were also identified as predictors—rs231775 CTLA4 in Random Forest and rs1800896 IL10 in both Random Forest and the C&RT model. However, these predictors were not decisive for statistically significant differences. This may be attributed to the limited number of observations, which represents a limitation of this study.

Given the numerous side effects of CNI therapy, efforts have been made to minimize or exclude their use from therapeutic regimens. In the absence of a better solution, research should focus on personalizing CNI therapy [85]. Determining single nucleotide polymorphisms (SNPs) is a crucial tool in developing a personalized approach to transplantation. Nevertheless, genome-wide multicenter association studies are needed to advance personalized transplant medicine [86].

The initial concept behind pharmacogenetics aimed to create a genetic ID for individual patients, allowing physicians to make optimal therapeutic decisions. Assuming that several genes directly affect drug metabolism in an exclusive way, this could be a milestone in therapy. Subsequent years of research have cast a shadow of uncertainty over this idea. Drug metabolism is influenced by many variable factors, not only genetic ones. Although we managed to demonstrate a strong relationship between the studied polymorphisms and CsA dosing, the study has certain limitations. As mentioned earlier, drug metabolism can be affected by various factors, including environmental conditions, diet, interactions with concomitant drugs, etc. Additionally, factors affecting the metabolism and bioavailability of vitamin D, such as the quality of supplementation and the accuracy of vitamin D concentration measurements during periodic check-ups, also play a role [87]. Creating a model that incorporates all of these variables exceeds the capabilities of our database. Moreover, accessing kidney transplant recipients is challenging due to their relatively small numbers compared to other patient groups, such as those with hypertension. It should be concluded that further, extensive, highly standardized multi-center studies are necessary, including much larger groups of patients. Data mining methods are an ideal analysis tool for this purpose.

## 5. Conclusions

The analysis using the random forest algorithm revealed that among other polymorphisms, both studied polymorphisms (rs2282679 *GC* and rs10741657 *CYP2R1*) had high predictive ranks for the C/D ratio-dependent variable. Among these, rs2282679 *GC* represented an overreaching predictive value in the C&RT model, whereas rs10741657 *CYP2R1* was not found to be predictive. Significant differences in cyclosporine C/D ratios were associated with combinations of rs2282679 *GC* and two other polymorphisms. Immune response-related polymorphisms were also identified as predictive by both algorithms; however, these did not result in statistically significant differences in cyclosporine C/D ratios. Precise pharmacogenomic profiling holds promise for enhancing the efficiency and safety of immunosuppressive therapy following kidney transplantation. Given the pleiotropic effects of vitamin D, the genetic factors influencing its metabolism are potentially important in this context.

## Figures and Tables

**Table 1:** Biochemical characteristics of the study group.

Variable	Mean	Median	Minimum	Maximum	Std. Dev.	Conf. SD −95%	Conf. SD +95%	Standard Error
WBC	7.67	7.32	1.85	17.31	2.43	2.20	2.71	0.18
RBC	4.23	4.21	2.86	5.53	0.59	0.53	0.65	0.04
HGB	12.83	12.80	8.80	17.10	1.70	1.54	1.90	0.13
HCT	37.89	37.80	27.00	51.40	4.81	4.35	5.37	0.36
PLT	222.11	213.00	45.00	467.00	71.46	64.69	79.82	5.39
Na	141.16	141.00	120.00	149.00	3.57	3.23	3.99	0.27
K	4.17	4.17	3.14	5.42	0.44	0.40	0.49	0.03
bilirubin	0.61	0.54	0.16	1.69	0.29	0.26	0.32	0.02
BUN	29.98	26.45	8.30	71.10	13.03	11.80	14.56	0.98
creatinine	1.61	1.44	0.67	4.31	0.64	0.58	0.71	0.05
uric acid	6.98	7.00	3.70	10.90	1.48	1.34	1.66	0.11
AlAT	19.33	16.00	3.00	73.00	11.15	10.09	12.45	0.84
AspAT	20.66	18.00	10.00	70.00	8.91	8.07	9.95	0.67
total CH	187.81	185.00	80.00	344.00	39.06	35.32	43.71	2.99
HDL	62.99	60.60	27.60	118.20	20.12	17.83	23.09	1.86
LDL	95.69	91.00	36.00	239.00	36.48	32.32	41.90	3.39
TG	142.87	127.00	48.00	404.00	66.46	58.90	76.26	6.14
lipids total	639.37	622.00	430.00	1183.00	133.75	118.53	153.49	12.37

**Table 2:** Clinical characteristics of the study group.

Variable	Mean	Median	Minimum	Maximum	Std. Dev.	Conf. SD −95%	Conf. SD +95%	Standard Error
Age [y]	54.27	55.00	22.00	82.00	12.41	11.23	13.85	0.93
Weight [kg]	78.89	78.00	40.00	139.00	17.25	15.62	19.27	1.30
Heihght [cm]	169.59	169.00	150.00	193.00	9.43	8.54	10.54	0.71
BMI [kg/m^2^]	27.34	26.37	17.31	42.90	5.12	4.63	5.71	0.38
Time from KTx [m]	10.38	10.00	1.00	27.00	6.24	5.65	6.97	0.47
Dose [mg]	171.30	150.00	50.00	500.00	61.27	55.48	68.41	4.60
Concentration [ng/mL]	116.37	113.00	2.00	315.90	48.03	43.49	53.63	3.61
Concentration/dose	0.72	0.67	0.01	1.75	0.28	0.25	0.31	0.02

**Table 4:** The differences in C/D ratio for CsA between particular genotypes in a post-hoc Tukey test. Statistical significances have been bolded.

Genotype	rs2282679GC CA rs1800896 IL10 AA (Leaf ID 5)	rs1800896 IL10 GG GA rs2032582 MDR2677bGT rs2282679 GC CC CA (Leaf ID 6)	rs602662 FUT2 AA rs2282679 GC (Leaf ID 9)
rs2282679 GC CA rs1800896 IL10 AA		*p* = 0.08	*p* = 0.54
**(Leaf ID 5)** rs1800896 IL10 GGGA rs2032582 MDR2677bGT	*p* = 0.08		***p*** = **0.006**
rs2282679 GC CC or CA **(Leaf ID 6)** rs602662 FUT2 AA	*p* = 0.57	***p*** = **0.006**	
rs2282679 GC **(Leaf ID 9)** rs602662 FUT2 GG AG rs1800896 IL10 AA **(Leaf ID 10)**	*p* = 0.62	*p* = 0.73	*p* = 0.08

## Data Availability

The data presented in this study are available on request from the corresponding author.
